# Content Validation of a Practice-Based Work Capacity Assessment Instrument Using ICF Core Sets

**DOI:** 10.1007/s10926-020-09918-7

**Published:** 2020-08-16

**Authors:** Johan H. Sengers, Femke I. Abma, Loes Wilming, Pepijn D. D. M. Roelofs, Yvonne F. Heerkens, Sandra Brouwer

**Affiliations:** 1 Department of Health Sciences, Community and Occupational Medicine, University of Groningen, University Medical Center Groningen, Groningen, The Netherlands; 2 Dutch Social Security Institute: Institute for Employee Benefits Schemes (UWV), Amsterdam, The Netherlands; 3 Research Centre for Insurance Medicine, AMC-UMCG-UWV-VUMC, Amsterdam, The Netherlands; 4 HAN University of Applied Sciences, Nijmegen, The Netherlands; 5 Dutch Institute of Allied Health Care, Amersfoort, The Netherlands

**Keywords:** Social security, Work capacity evaluation, Biopsychosocial, Disability evaluation, Participation, ICF

## Abstract

**Purpose:**

A shift from providing long-term disability benefits to promoting work reintegration of people with remaining work capacity in many countries requires new instruments for work capacity assessments. Recently, a practice-based instrument addressing biopsychosocial aspects of functioning, the Social Medical Work Capacity instrument (SMWC), was developed. Our aim was to examine the content validity of the SMWC using ICF core sets.

**Methods:**

First, we conducted a systematic search to identify relevant ICF core sets for the working age population. Second the content of these core sets were mapped to assess the relevance and comprehensiveness of the SMWC. Next, we compared the content of the SMWC with the ICF-core sets.

**Results:**

Two work-related core sets and 31 disease-specific core sets were identified. The SMWC and the two work-related core sets overlap on 47 categories. Compared to the work-related core sets, the Body Functions and Activities and Participation are well represented in the new instrument, while the component Environmental factors is under-represented. Compared to the disease-specific core sets, items related to the social and domestic environmental factors are under-represented, while the SMWC included work-related factors complementary to the ICF.

**Conclusion:**

The SMWC content seems relevant, but could be more comprehensive for the purpose of individual work capacity assessments. To improve assessing relevant biopsychosocial aspects, it is recommended to extend the instrument by adding personal and environmental (work- and social-related) factors as well as a more tailored use of the SMWC for assessing work capacity of persons with specific diseases or underlying illness.

**Electronic supplementary material:**

The online version of this article (10.1007/s10926-020-09918-7) contains supplementary material, which is available to authorized users.

## Background

The increasing rates of long-term sickness absence and work disability in an ageing population have obliged several countries to shift their focus from providing long-term disability benefits and social protection programmes to promoting the work reintegration of people with partial or residual work capacity [1–3]. By introducing policy reforms, many countries have shifted their focus away from assessing disability on predominantly medical grounds to the assessment of the remaining work capacity of disability benefit claimants [4].

Several countries have developed new assessment instruments over the last ten years to assess individuals’ abilities to participate in the labour market actively, to assess barriers which may restrict work participation, and to indicate directions interventions may take to overcome barriers for work participation [4, 5]. These new assessment instruments have shifted from predominantly focusing on loss of physical and/or mental functioning, towards assessment of work capacity from a holistic perspective, i.e. the ability to participate actively in the labour market from physical, mental, social, and societal perspectives. Instead of the traditional disability assessment instruments, new instruments should not only assess limitations in activities [6], but also incorporate personal and environmental factors [1] which could mitigate limitations in activities when appropriate adjustments are applied. Although a biopsychosocial approach [3, 4, 6] has been integrated into many of these instruments, the literature about the validity of these instruments is limited.

Recognizing the interaction of activity limitations with the particular requirements of the individual’s work context led to the development of a novel approach for work capacity assessments by the Dutch Social Security Institute, the Institute for employee benefits schemes (UWV). The Social Medical Work Capacity instrument, SMWC, was developed by a panel of experts of the UWV (e.g. staff members, labour experts, and insurance physicians) and based on the International Classification of Functioning, Disability and Health (ICF) [7]. It has been developed to help the UWV professionals asssessing a clients’ ability to participate in work and to provide indications and/or advice for reintegration support to optimize the use of available potential and finding a good jobmatch [6, 8, 9]. The instrument was pilot tested in practice and showed that professionals using the instrument were positive as it provides a structure for describing the clients work capacity and their possibilities to participate in work [10, 11]. However, the professionals also critizised the large amount of items making the instrument timely in its use. Providing a better evidence base for the content of the instrument could improve utility of this new instrument in practice. Therefore, insight is needed whether all included items are relevant.

To examine the relevance of items needed to assess claimants’ remaining work capacity, it is important to evaluate the content validity of the SMWC, i.e. the degree to which the content of the instrument is an adequate reflection of the construct to be measured [12, 13], and to evaluate whether all items are relevant and comprehensive for the construct to be measured [14]. To validate the content of the SMWC, ICF core sets can be of potential benefit to determining these factors. They provide a minimum standard for the assessment and reporting of functioning and health [15]. Each ICF core set includes a selection of essential categories from the full ICF classification considered most relevant for describing the functioning and environmental factors of a person with a specific health condition or in a specific healthcare context. ICF core sets are frequently used in daily practice by clinicians and other professionals for the assessment and reporting of functioning and health [15].

The overall aim of the present study was to examine the content validity of SMWC by comparing the content of the instrument with ICF core sets.

## Method

### The Social Medical Work Capacity instrument (SMWC)

The Social Medical Work Capacity instrument, SMWC, was developed by a group of experts working as professionals at the Dutch Social Security Institute, the Institute for employee benefits schemes (UWV). The instrument is designed to guide social security professionals in taking a biopsychosocial approach when creating an overview of a person’s work capacity and what is needed to find a good jobmatch [8, 9]. The 129 items of SMWC related to 95 2nd level ICF categories, of which 54 from the Body Functions, 35 from Activities and Participation, and six from Environmental factors. With the exception of Chapter 6 (domestic life) and Chapter 9 of Activities and Participation (Community, social and civic life), all Chapters of the Classification of Body Functions and the Classification of Activities and Participation are represented in the SMWC. The SMWC does not include categories from the Classification of Body Structures. The ICF categories of the Classification of Environmental factors are mostly related to the work environment, such as climate, light, sound, vibration, and air quality (Chapter 1 and 2). Some ICF categories were further specified in the SMWC to provide more detail of work capacity items which is needed to exploit this capacity in actual work. Supplementary Table S2 presents a full overview of included ICF categories in the SMWC.

#### Procedure

First, we conducted a systematic search to identify relevant articles on relevant core sets for the working age population. Second the content of these core sets were mapped to the content of the SMWC. Next, we compared the content of the SMWC with the ICF-core sets.

### ICF Core Sets

Medline, PsycINFO (both using Ebsco), and Web of Science were searched using the terms ‘disability evaluation’, ‘work capacity’, and ‘work ability’ combined with search terms identifying assessment instruments (including questionnaires) and ICF core sets [15]. The full search strategy can be found in Supplementary Table S1. The databases were searched for articles published between January 2000 and July 2018. Although the ICF was published in 2001, the year 2000 was also included as there was already a draft version of the ICF available.

### Selection of Articles

Articles were included if they described the development of an ICF core set and presented final results. Letters to the editor, guidelines, editorials, book Chapters, dissertations, conference proceedings, design papers or case reports were excluded. Core sets were included if they were designed for the assessment of the functioning of working age (18–65) people with a specific disease or for their assessment in a work-related setting. A core set was excluded if the context precluded work, e.g. in acute or post-acute settings and geriatric settings. Additionally, core sets were excluded if they were developed in a too specific setting (e.g. applicable in a specific country). The ICF Research Branch website was checked for completeness [16]. A first selection based on title and abstract was conducted by two independent reviewers. When the reviewers could not reach consensus, a third reviewer was consulted. When the title and abstract did not provide enough information to decide if the inclusion criteria were met, the article was included for full-text screening. Disease-specific core sets are grouped into disease groups in line with the ICD-10 [17] and in accordance with the most prevalent diseases of people claiming disability benefits.

### Data Extraction

**Fig. 1:**
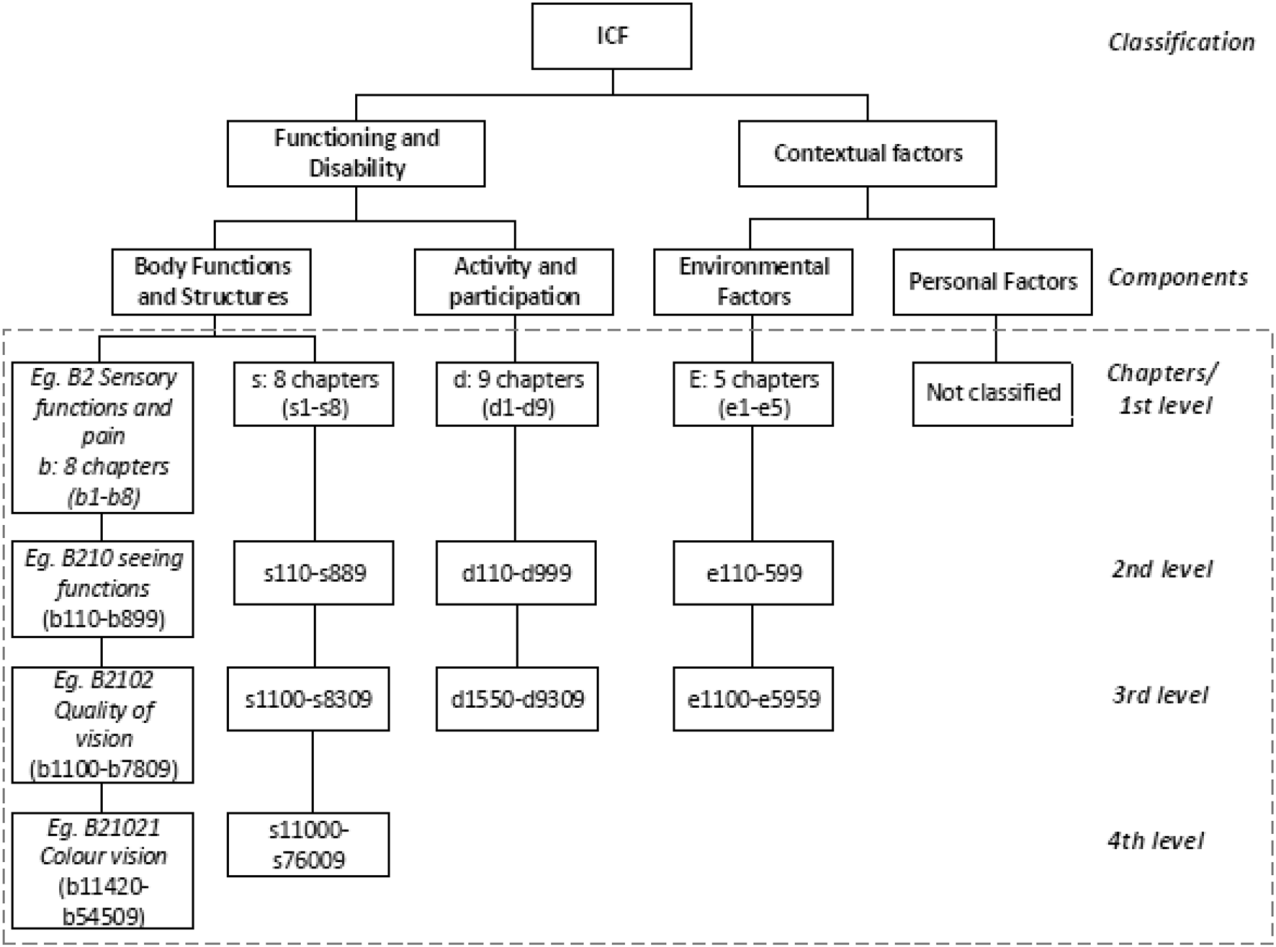
The hierarchical structure of the ICF

First, data regarding core set, study aim, number of ICF categories included, and methods used were extracted from the included full-text articles by two reviewers. The methodological quality of the core set development was described taking ‘the guide on how to develop an ICF core set’ by Selb et al. [15] as the gold standard. When this gold standard was not applied in the development, the method used was described. Second, all ICF categories included in the core sets were registered. Data extraction was limited to the second level order of ICF categories. Figure 1 shows the hierarchical structure of the ICF classification. To allow for comparison with ICF core sets, the SMWC was compared on 2nd level ICF categories, collapsing items of 3rd and 4th level under the related 2nd level category.

To structure the results, the included 31 disease-specific core sets were grouped into disease groups in line with the ICD-10 [17] and in accordance with the most prevalent diseases of people claiming disability benefits [18, 19]. Two work-related core sets completed the total inclusion of 33 core sets.

### Content Comparison of SMWC with ICF Categories

First, to evaluate whether the SMWC contains all relevant items for the purpose of holistic work capacity assessments, a comparison was made with the two work-related core sets as these core sets are related to the construct of the SMWC.

Second, to evaluate whether the SMWC is comprehensive and thus covers all relevant aspects of the construct to be measured and whether all included items of the SMWC are relevant for the construct, we compared the content of the SMWC to the content of all retrieved ICF core sets, going beyond a specific work-related focus. This allowed for comparing the SMWC with core sets developed for reporting on functioning and health, and may lead to identification of common indicators across disease specific core sets which are possibly relevant to include in the new instrument. We used a relevance ranking by calculating the relative frequency of each ICF category within the disease groups. Scores of 0% indicated that an ICF category was not included in any core set, and scores of 100% indicated that an ICF category was included in all core sets of that particular disease group. Presentation of this relevance ranking was restricted to scores higher than 70%, a rather arbitrary cut-off. Subsequently, these common indicators were compared to the content of the work capacity instrument.

## Results

### Search

**Fig. 2:**
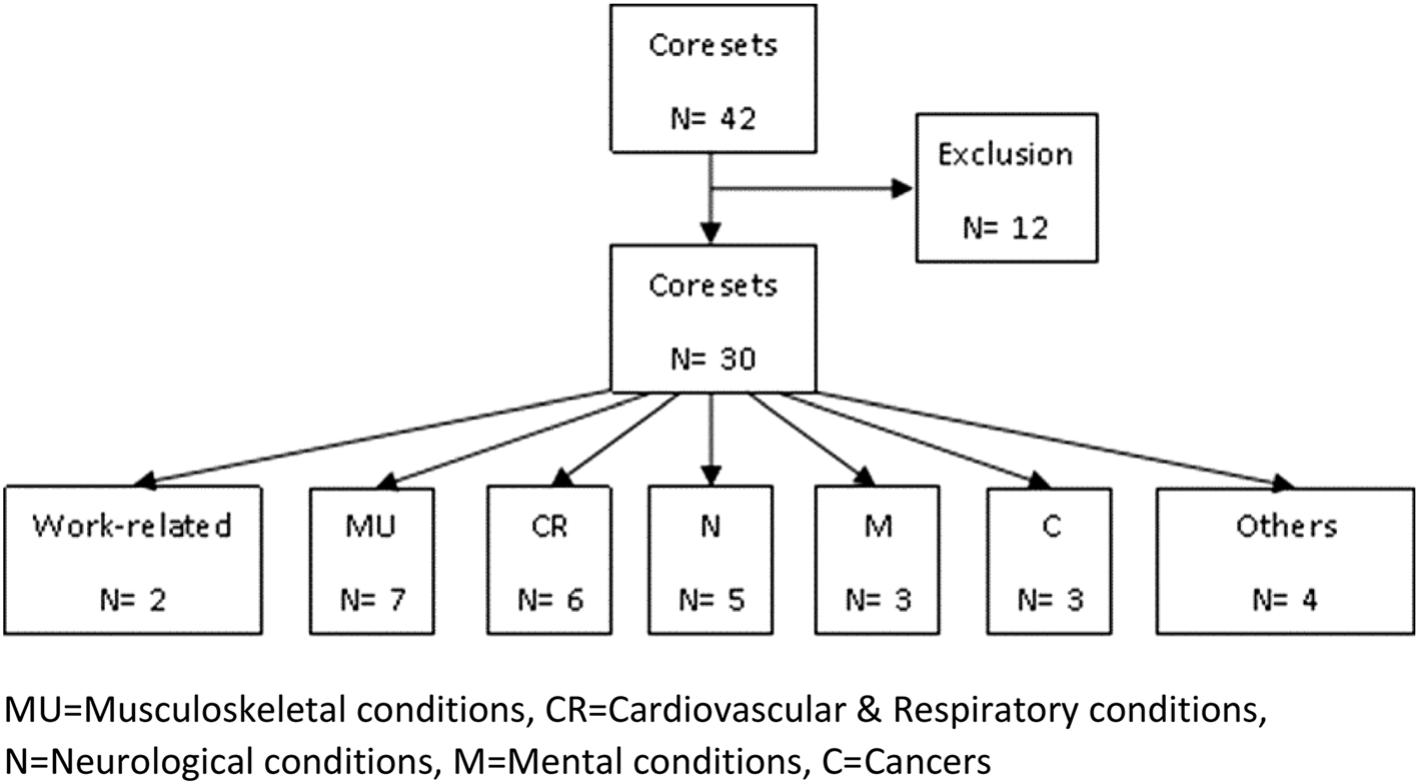
Flowchart of ICF core set inclusion

The combined searches yielded 3376 hits (1950 in Medline/PubMed, 637 in PsycINFO, 789 in Web of Science). After removal of duplicates, a total of 2267 abstracts were identified and 277 full-text articles on core sets were read. Forty-five articles described the development of a core set and presented the final results. Of these, 33 articles met the inclusion criteria and were included. A reference check of the included articles and a check of the ICF Research Branch website did not identify additional articles or core sets. However, our search retrieved three core sets that were not included on the website. Figure 2 depicts how the core sets were selected, and Table 1 provides a description of the characteristics of the included articles and two work-related and 31 disease specific core sets.

**Table 1:** Overview of included articles and their core sets, aggregated by disease group

Authors, year	Title	ICF core set	Aim of study	No. of ICF categories	Methods
Musculoskeletal conditions					
Boonen et al., 2010 [32]	ASAS/WHO ICF Core Sets for ankylosing spondylitis (AS): how to classify the impact of AS on functioning and health	Core Sets for AS	To report on the results of a standardized consensus process agreeing on concepts typical of and/or relevant when classifying functioning and health in patients with AS based on the International Classification of Functioning and Health (ICF)	80	^a^
Cieza et al., 2004 [33]	ICF Core Set for chronic widespread pain (CWP)	Core Sets for CWP	To develop the first versions of a comprehensive and a brief ICF core set for CWP	67	^d^
Cieza et al., 2004 [34]	ICF Core Sets for low back pain (LBP)	Core Sets for LBP	To report on the results of the consensus process integrating evidence from preliminary studies to develop the first versions of a comprehensive and brief ICF core set for LBP	78	^d^
Cieza et al., 2004 [35]	ICF Core Sets for Osteoporosis (OP)	Core Sets for OP	To report on the results of the consensus process integrating evidence from preliminary studies to develop the first versions of a comprehensive and a brief ICF core set for OP	69	^d^
Dreinhofer et al., 2004 [36]	ICF Core sets for Osteoarthritis (OA)	ICF Core Sets for OA	To report on the results of the consensus process integrating evidence from preliminary studies to develop the first versions of a comprehensive and a brief ICF core set for OA	55	^d^
Grill et al., 2007 [37]	International Classification of Functioning, Disability and Health (ICF) Core Set for patients with acute arthritis	Core Sets for patients with acute arthritis	To construct a preliminary version of the ICF core set for acute inflammatory arthritis	79	^e^
Stucki et al., 2004 [38]	ICF Core Sets for Rheumatoid Arthritis (RA)	Core Sets for RA	To report on the results of the consensus process integrating evidence from preliminary studies to develop the first versions of a comprehensive and a brief ICF core set for RA	96	^d^
Cardiovascular and respiratory conditions					
Cieza et al., 2004 [39]	ICF Core Set for chronic ischaemic heart disease (CIHD)	Core Sets for CIHD	To develop the first versions of a comprehensive and a brief ICF core set for CIHD	61	^d^
Geyh et al., 2004 [40]	ICF Core Sets for Stroke	Core Sets for Stroke	To report on the results of the consensus process integrating evidence from preliminary studies to develop the first versions of a comprehensive and a brief ICF core set for stroke	130	^d^
Ruof et al., 2004 [41]	ICF Core Sets for diabetes mellitus (DM)	Core Sets for DM	To develop the first versions of a comprehensive and a brief ICF core set for DM	99	^d^
Stucki et al., 2004 [42]	ICF Core Sets for Obesity	ICF Core Set for Obesity	To report on the results of the consensus process integrating evidence from preliminary studies to develop the first versions of a comprehensive and a brief ICF core set for obesity	109	^d^
Stucki et al., 2004 [43]	ICF Core sets for obstructive pulmonary diseases (OPD)	ICF Core Set for OPD	To report on the results of the consensus process integrating evidence from preliminary studies to develop the first versions of a comprehensive and a brief ICF core set for OPD	71	^d^
Viehoff et al., 2015 [44]	Development of consensus International Classification of Functioning, Disability and Health (ICF) core sets for lymphedema	ICF Core Set for Lymphedema	To present the outcomes of a consensus conference held to determine the first version of an ICF core sets for lymphedema	43	^b^
Neurological conditions					
Cieza et al., 2010 [45]	ICF Core Sets for individuals with spinal cord injury (SCI) in the long-term context	Core Sets for SCI	To report on the results of the consensus process to develop the first versions of a comprehensive and a brief ICF core set for individuals with SCI in the long-term context	168	^b^
Coenen et al., 2011 [46]	The development of ICF Core Sets for multiple sclerosis (MS): results of the International Consensus Conference	Core Set for MS	To report on the results of an evidence-based International Consensus Conference to develop a comprehensive and a brief ICF core set for MS	138	^b^
Gradinger et al., 2011 [47]	Part 1.ICF Core Sets for people with sleep disorders	Core Set for Sleep Disorder	To report on the results of the consensus process in developing a comprehensive and brief ICF core set for sleep disorders	120	^b^
Khan and Pallant, 2011 [48]	Use of the ICF to identify preliminary comprehensive and brief core sets for Guillain Barre syndrome (GBS)	Core Set for GBS	To develop the first versions of a comprehensive and a brief ICF core set for GBS	99	^c^
Laxe et al., 2013 [49]	Development of the International Classification of Functioning, Disability and Health core sets for traumatic brain injury (TBI): An International consensus process	Core Set for TBI	A formal decision-making and consensus process is presented to develop the first versions of an ICF core set for TBI	139	^b^
Mental conditions					
Ayuso-Mateos et al., 2013 [50]	Development of the International Classification of Functioning, Disability and Health core sets for bipolar disorders: results of an international consensus process	Core Sets for Bipolar Disorders	To describe the process of the development of two core sets for bipolar disorder (BD) in the framework of the ICF	38	^g^
Brütt et al., 2013 [51]	Development of an ICF-based core set of activities and participation for patients with mental disorders: an approach based upon data	Activities and participation for patients with mental disorders	To identify relevant ICF categories of the Activities and Participation component for a core set for adult patients with mental disorders (A&P ICF-MD). Other components were excluded	27	^f^
Cieza et al., 2004 [52]	ICF Core Set for depression	Core Sets for depression	To develop the first versions of a comprehensive and a brief ICF core set for depression	121	^d^
Gomez-Benito et al. 2017 [53]	Beyond diagnosis: the Core Sets for persons with schizophrenia based on the ICF	Core set for Schizophrenia	To develop the first version of the comprehensive and brief ICF core set for schizophrenia	97	^a^
Cancers					
Brach et al., 2004 [54]	ICF Core sets for breast cancer	Core Sets for breast cancer	To develop the first versions of a comprehensive and a brief ICF core set for breast cancer	80	^d^
Geerse et al., 2016 [55]	Health-related problems in adult cancer survivors: development and validation of the Cancer Survivor Core Set	Core Set for Cancer Survivors	To develop and validate the Cancer Survivor Set	19	^h^
Tschiesner et al., 2010 [56]	Development of ICF core sets for Head and Neck cancer	Core Set for Head and Neck cancer	To develop the first version of an ICF core set for Head and Neck Cancer	112	^g^
Other diseases					
Bölte et al., 2018 [57]	Standardised assessment of functioning in ADHD: consensus on the ICF Core Sets for ADHD	Core Sets for ADHD	To develop a comprehensive, a common brief and three age-appropriate brief ICF Core Sets for ADHD	72	^a^
Bölte et al.,2018 [58]	The Gestalt of functioning in autism spectrum disorder:Results of the international;l conference to develop final consensus of ICF core sets	Core Sets for autism spectrum disorder	To identify a comprehensive, a common brief, and three age-appropriate brief autism spectrum disorder Core Sets	111	^a^
Danermark et al., 2013 [59]	The Creation of a Comprehensive and a Brief Core Set for Hearing Loss Using the International Classification of Functioning, Disability and Health	Core sets for Hearing Loss	To describe the creation of comprehensive and brief core sets for Hearing Loss	117	^b^
Grill et al., 2012 [60]	ICF Core Set for patients with vertigo, dizziness and balance disorders	Core Sets for Vertigo and Dizziness	To develop ICF core sets for patients with vertigo and dizziness to describe functioning	100	^g^
Peyrin-Biroulet et al., 2012 [61]	Development of the first disability index for inflammatory bowel disease (IBD) based on the international classification of functioning, disability and health	Core Set for IBD	To develop the first disability index for IBD by selecting the most relevant ICF categories affected by IBD	36	^b^
Rudolf et al., 2012 [62]	Development of the international classification of functioning, disability and health core sets for hand conditions − results of the world health organization international consensus process	Core Set for Hand Condition	To develop the first version of an ICF core set for Hand Conditions	117	^a^
Work related					
Brage et al., 2008 [63]	Development of ICF core set for disability evaluation in social security	Core Set for disability evaluation in social security	To report on the development of an ICF core set for functional assessment in disability claims in European social security systems. Environmental factors are not included cause of insufficient support in the consensus process	20	^i^
Finger et al., 2012 [64]	ICF Core Set for vocational rehabilitation (VR): results of an international consensus conference	ICF Core Set for VR	To present an ICF core set for VR with the specific aim of describing the consensus process and presenting the lists of categories for the core set	90	^a^

^a^‘The guide on how to develop an ICF core set’ from Selb et al. [15] involves two steps. First, four preparatory studies should be conducted: a systematic literature review, a qualitative study, an expert survey and an empirical multicentre study. Second, an international consensus conference is organized using the results of the preparatory studies as a starting point for a structured decision-making process. If at least 75% of the participants achieve consensus regarding an ICF category, it is included in the core set. Less than 40% consensus means exclusion. Consensus decisions between 40 and 74% are discussed in plenary and a cut off of 50% agreement is applied

^b^According Selb et al. (a), but exact decision-making and consensus cut-off percentages are not revealed

^c^Similar to (a) but with three preliminary studies and no separate patient perspective involved. The consensus process consists of three rounds. In the first round less than 50% consensus means exclusion. After several voting rounds and discussion, the final plenary session also features a cut off of 50% agreement

^d^The preparatory phase consisted of three preliminary studies. No patient perspective by qualitative study is included. The exact decision-making and consensus cut-off percentages are not revealed

^e^Similar to (c) but instead of a survey of the health professionals, they conducted focus groups

^f^Three preliminary studies. First, a content analysis of the relevant outcome instruments were identified in a systematic review. Second, focus groups including rehabilitation patients were used to corroborate and complement the findings from the outcome instrument content analysis. Third, an expert panel selected activities and participation categories identified in steps one and two according to their relevance to clinical practice. Finally, the categories for inclusion in the A&P ICF-MD were defined, based on formal decision procedures

^g^Similar to (a) but patient perspective was included by semi-structured interviews instead of focus groups

^h^Delphi study which involved patients, medical experts and healthcare workers. Categories were selected from all second-level ICF categories. Decision-making and consensus process conducted in two rounds, independently and anonymously and with no discussion. Validation by questionnaire selection and linking procedure

^i^A formal decision-making process was applied. First, national meetings with experts suggested the categories to be included in the core set. The members of the EUMASS working group for the ICF then selected a core set based on these suggestions by a formal voting procedure. In the first voting round > 80% agreement was included, < 20% excluded. All scores in-between were discussed in a second round. Inclusion in the second voting round needed 50% or more agreement

### Content Comparison of SMWC with ICF Categories

#### Work-Related Core Sets

The two work-related core sets are the Vocational Rehabilitation core set and the Disability Evaluation core set (see Text Box 1 for a further description). The SMWC, existing of 129 categories, and the two work-related core sets overlap on 47 categories (36% of the SMWC), mainly in Chapters Mental functions (b1), Learning and applying knowledge (d1), General tasks and demands (d2), Mobility (d4), Interpersonal interactions (d7), and Natural environment and human made changes to environment (e2) (see Table 2; Supplemental Figure S1). As well as the SMWC, both work-related core sets do not include categories from the Classification of Body Structures. The SMWC overlaps on 17 categories with the Disability Evaluation core set (13.2% of the SMWC) and 46 with the Vocational Rehabilitation core set (35.7% of the SMWC). A total of 54 ICF categories are included in the SMWC but not in any work-related core set (41.9% of the SMWC), of which N = 37 are from the Body functions, reflecting mainly physical and mental functions. In turn, the work related core sets contain 44 ICF categories not included in the SMWC, with the majority from the component of Environmental factors (N = 28). For instance, the Vocational Rehabilitation core set includes Environmental factors within the four ICF Chapters Products and technology (e1), Support and relationships (e3), Attitudes (e4), and Services, systems and policies (e5), which are all not included in SMWC (see Table 2). The Disability Evaluation core set does not include any Environmental factors because no consensus could be reached during its development on which factor to include [11]. See Supplementary Fig. 1 for an overview of overlap between the SMWC and the two work-related core sets on the ICF components (Table 3).

**Table 2:** Content comparison SMWC and work-related core-sets

Body functions		DE	VR	SMWC	Body functions		DE	VR	SMWC	Body functions		DE	VR	SMWC	
**b1**	Mental functions				**b2**	Sensory functions and pain				b5	Functions of the digestive, metabolic and endocrine systems				
b110	Consciousness functions			x	*b210*	*Seeing functions*		*x*	*x*	b525	Defecation functions			x	
*b114*	*Orientation functions*		*x*	*x*	*b230*	*Hearing functions*		*x*	*x*	b540	General metabolic functions			x	
b117	Intellectual functions			x	*b235*	*Vestibular functions*		*x*	*x*	b550	Thermoregulatory functions			x	
b122	Global psychosocial functions			x	b240	Sensations associated with hearing and vestibular function			x	b555	Endocrine gland functions			x	
*b125*	*Dispositions and intra-personal functions CY*		*x*	*x*	b260	Proprioceptive functions			x	**b6**	Genitourinary and reproductive functions				
*b126*	*Temperament and personality functions*		*x*	*x*	b265	Touch function			x	b620	Urination functions			x	
*b130*	*Energy and drive functions*		*x*	*x*	b270	Sensory functions related to temperature and other stimuli			x	**b7**	Neuromusculoskeletal and movement-related functions				
b134	Sleep functions		x		*b280*	*Sensation of pain*	*x*	*x*	*x*	*b710*	*Mobility of joint functions*	*x*		*x*	
*b140*	*Attention functions*		*x*	*x*	**b3**	Voice and speech functions				b715	Stability of joint functions			x	
b144	Memory functions			x	b310	Voice functions			x	*b730*	*Muscle power functions*	*x*	*x*	*x*	
*b147*	*Psychomotor functions*		*x*	*x*	b320	Articulation functions			x	b735	Muscle tone functions			x	
b152	Emotional functions			x	b330	Fluency and rhythm of speech functions			x	*b740*	*Muscle endurance functions*		*x*	*x*	
*b156*	*Perceptual functions*		*x*	*x*	**b4**	Functions of the cardiovascular, hematological, immunological and respiratory systems				b750	Motor reflex function			x	
b160	Thought functions			x	b410	Heart functions			x	b755	Involuntary movement reaction functions			x	
*b163*	*Basic cognitive functions*	*x*	*x*	*x*	b415	Blood vessel functions			x	b760	Control of voluntary movement functions			x	
b164	Higher level cognitive functions			x	b420	Blood pressure functions			x	b765	Involuntary movement functions			x	
b167	Mental functions of language			x	b430	Hematological system functions			x	**b8**	Functions of the skin and related functions				
b172	Calculation functions			x	b435	Immunological system functions			x	*b810*	*Protective functions of the skin*		*x*	*x*	
b176	Mental functions of			x	b440	Respiration functions			x	b820	Repair functions of the skin			x	
	sequencing complex				b445	Respiratory muscle function			x						
	movements				b450	Additional respiratory functions			x						
					*b455*	*Exercise tolerance functions*	*x*	*x*	*x*						

Activities and participation		DE	VR	SMWC	Activities and participation		DE	VR	SMWC	Activities and participation		DE	VR	SMWC	
d1	Learning and applying knowledge				**d3**	Communication				**d5**	Self-care				
d110	Watching	x		x	d310	*Communicating with-receiving- spoken messages*		x	x	d510	Washing oneself			x	
d115	Listening	x		x	d315	*Communicating with-receiving- non-verbal messages*		x	x	d520	Caring for body parts			x	
d120	Other purposeful sensing			x	d325	Communicating with-receiving-written messages			x	d530	Toileting		x	*x*	
d155	Acquiring skills	x	x	x	d330	Speaking			x	d540	Dressing		x	*x*	
					d335	Producing non-verbal messages			x	d570	Looking after one’s health		x		
d159	Basic learning, to remind			x	d340	Producing messages in formal sign language			x	d598	Self-care, safety			x	
*d160*	*Focusing attention*		*x*	*x*	d345	Writing messages			x						
d163	Thinking		x		d349	Communication-producing, expressing own feelings			x	**d7**	Interpersonal interactions and relationships				
*d166*	*Reading*		*x*	*x*	d350	Conversation		x		*d710*	*Basic interpersonal interactions*		*x*	*x*	
*d170*	*Writing*		*x*	*x*	d360	Using communication devices and techniques		x		*d720*	*Complex interpersonal interactions*	*x*	*x*	*x*	
*d172*	*Calculating*		*x*	*x*	d399	Communication, unspecified	x			d730	Relating with strangers			x	
d175	Solving problems		x		**d4**	Mobility				*d740*	*Formal relationships*		*x*	*x*	
d177	Making decisions	x	x		*d410*	*Changing basic body position*	*x*	*x*	*x*	**d8**	Major life areas				
**d2**	General tasks and demands				*d415*	*Maintaining a body position*	*x*	*x*	*x*	d820	School education		x		
*d210*	*Undertaking a single task*		*x*	*x*	*d430*	*Lifting and carrying objects*	*x*	*x*	*x*	d825	Vocational training		x		
*d220*	*Undertaking multiple tasks*	*x*	*x*	*x*	*d435*	*Moving objects with the lower extremities*				d830	Higher education		x		
*d230*	*Carrying out daily routine*		*x*	*x*	*d440*	*Fine hand use* Use keyboard	*x*	*x*	*x*	d840	Apprenticeship (work preparation)		x		
*d240*	*Handling stress and other psychological demands*	*x*	*x*	*x*	*d445*	*Hand and arm use*	*x*	*x*	*x*	d845	Acquiring, keeping and terminating a job		x		
d250	Managing one's own behavior CY			x	*d450*	*Walking*	*x*	*x*	*x*	d850	Remunerative employment		x		
d298	General tasks and demands, Estimating own options Overseeing the actions of own actions Achieving workpace			x	*d455*	*Moving around*		*x*	*x*	d855	Non-remunerative employment		x		
					d465	Moving around using equipment		x		d870	Economic self-sufficiency		x		
					d469	Walking and moving, Fine foot use			x	d859	Work and employment Number of hours you can work per day/week Handling different types of working hours Level of exertion			x	
					*d470*	*Using transportation*	*x*	*x*	*x*						
					*d475*	*Driving*		*x*	*x*						

Environmental factors		DE	VR	SMWC	Environmental factors		DE	VR	SMWC	Environmental factors		DE	VR	SMWC	
**e1**	Products and technology				**e3**	Support and relationships				**e5**	Services, systems and policies				
e110	Products or substances for personal consumption		x		e310	Immediate family		x		e525	Housing services, systems and policies		x		
e115	Products and technology for personal use in daily living		x		e320	Friends		x		e535	Communication services, systems and policies		x		
e120	Products and technology for personal indoor and outdoor mobility and transportation		x		e325	Acquaintances, peers colleagues, neighbors and community members		x		e540	Transportation services, systems and policies		x		
e125	Products and technology for communication		x		e330	People in positions of authority		x		e550	Legal services, systems and policies		x		
e130	Products and technology for education		x		e340	Personal care providers and personal assistants		x		e555	Associations and organizational services, systems and policies		x		
*e135*	*Products and technology for employment* Exposure to specific substances Wear of protective equipment		*x*	*x*	e355	Health Professionals		x		e565	Economic services, systems and policies		x		
e150	Design, construction and building products and technology of buildings for public use		x		e360	Health related professionals		x		e570	Social security services, systems and policies		x		
e155	Design, construction and building products and technology of buildings for private use		x		**e4**	Attitudes				e580	Health services, systems and policies		x		
**e2**	Natural environment and human-made changes to environment				e430	Individual attitudes of people in positions of authority		x		e585	Education and training services, systems and policies		x		
*e225*	*Climate* Temperature, heat Temperature, cold		*x*	*x*	e445	Individual attitudes of strangers				e590	Labor and employment services, systems and policies		x		
*e240*	*Light*		*x*	*x*	e450	Individual attitudes of health professionals		x							
*e250*	*Sound*		*x*	*x*	e460	Societal attitudes		x							
e255	Vibration			x	e465	Social norms, practices and ideologies		x							
*e260*	*Air quality*		*x*	*x*											

ICF categories presented in italic present overlap in SMWC and work-related core sets

*SMWC* Social Medical Work Capacity instrument, *DE* Disability Evaluation core-set, *VR* Vocational Rehabilitation core-set

**Table 3:** Relevance ranking ICF categories in the disease specific core sets

ICF category		MU	CR	N	M	C	Mean
**b152**	**Emotional functions**	100	100	100	75	100	95
e310	Immediate family	100	100	100	75	100	95
e355	Health professionals	100	100	100	75	100	95
e410	Individual attitudes of immediate family members	100	83	100	75	100	92
e580	Health services, systems and politics	100	83	100	75	100	92
**d240**	**Handling stress and other psychological demands**	71	83	100	100	100	91
d920	Recreation and leisure	86	100	100	100	67	91
d770	Intimate relationships	100	100	80	100	67	89
**b130**	**Energy and drive functions**	86	83	100	75	100	89
e110	Products or substances for personal consumption	100	100	100	75	67	88
**b280**	**Sensation of pain**	86	100	100	50	100	87
d570	Looking after one’s health	57	100	80	100	100	87
e570	Social security services, systems and policies	86	67	100	75	100	86
e320	Friends	71	100	80	75	100	85
d640	Doing housework	100	83	100	75	67	85
b640	Sexual functions	57	83	100	75	100	83
**d475**	**Driving**	86	83	100	75	67	82
d850	Remunerative employment	100	100	100	75	33	82
**d230**	**Carrying out daily routine**	57	67	100	100	67	78
e450	Individual attitudes of health professionals	100	83	100	75	33	78
**d510**	**Washing oneself**	100	67	80	75	67	78
e460	Societal attitudes	86	83	100	75	33	75
**d470**	**Using transportation**	100	67	100	75	33	75
d620	Acquisition of goods and services	100	83	80	75	33	74
d760	Family relationships	57	67	80	100	67	74
e340	Personal care providers and personal assistants	71	100	80	50	67	74
e420	Individual attitudes of friends	71	100	80	75	33	72
d845	Acquiring, keeping and terminating a job	57	67	100	100	33	71
b134	Sleep functions	14	100	100	75	67	71
**d540**	**Dressing**	100	67	80	75	33	71
**b455**	**Exercise tolerance functions**	86	100	100	0	67	71

All 2nd level ICF categories, resented in percentages in the grouped core sets for each disease group, truncated at 70% level. Full results available upon request by authors. ICF categories also present in SMWC are presented in **Bold**

*Mean* mean across the five groups, *MU* Musculoskeletal conditions, *CR* Cardiovascular & Respiratory conditions, *N* Neurological conditions, *M* Mental conditions, *C* Cancers, *SMWC* Social Medical Work Capacity instrument

Text Box 1: Work-Realted Core Sets Two work-related core sets were identified: the EUMASS core set for Disability Evaluation (DE) [11] and the Vocational Rehabilitation (VR) core set [12]. Both work-related core sets have a specific focus and possibilities for use in assessing work capacity. The **Disability Evaluation core** set is a generic tool for medical advisors in social security to help them in taking decisions such as assessment of work disability claims and to improve quality of decisions and inter-professional communication. However, due to the lack of consensus, no environmental factors were included. It includes 20 ICF categories useful for work disability evaluation, with the majority from activities and participation (N = 15) and body functions (N = 5). The **Vocational Rehabilitation core set** is aims to guide implementing rehabilitation programs for individuals of working age with restricted work participation due to disease, injury, or a health-related event. It consists of 90 ICF categories, with the majority from the activities and participation (N = 40) and environmental factors component (N = 33). Both core sets are generic, i.e., applicable to all cases regardless of diagnosis. Although in social security settings each disability assessment usually starts with examining a medical report with the main diagnosis. Together the two work-related core sets contain 94 2nd level ICF categories from Body Functions (N = 18), Activities and Participation (N = 43) and Environmental factors (N = 33)

#### Disease-Specific Core Sets

The 33 disease-specific core sets were grouped into musculoskeletal conditions, cardiovascular and respiratory conditions, neurological conditions, mental conditions, and cancers, see Text Box 2 for a further description and grouping. First, when looking at the distribution of included ICF categories across the disease groups, the ICF categories are more or less equally divided over the Body Functions, Activities and Participation and Environmental factors, while 7.4% are from the Body structures.

**Fig. 3:**
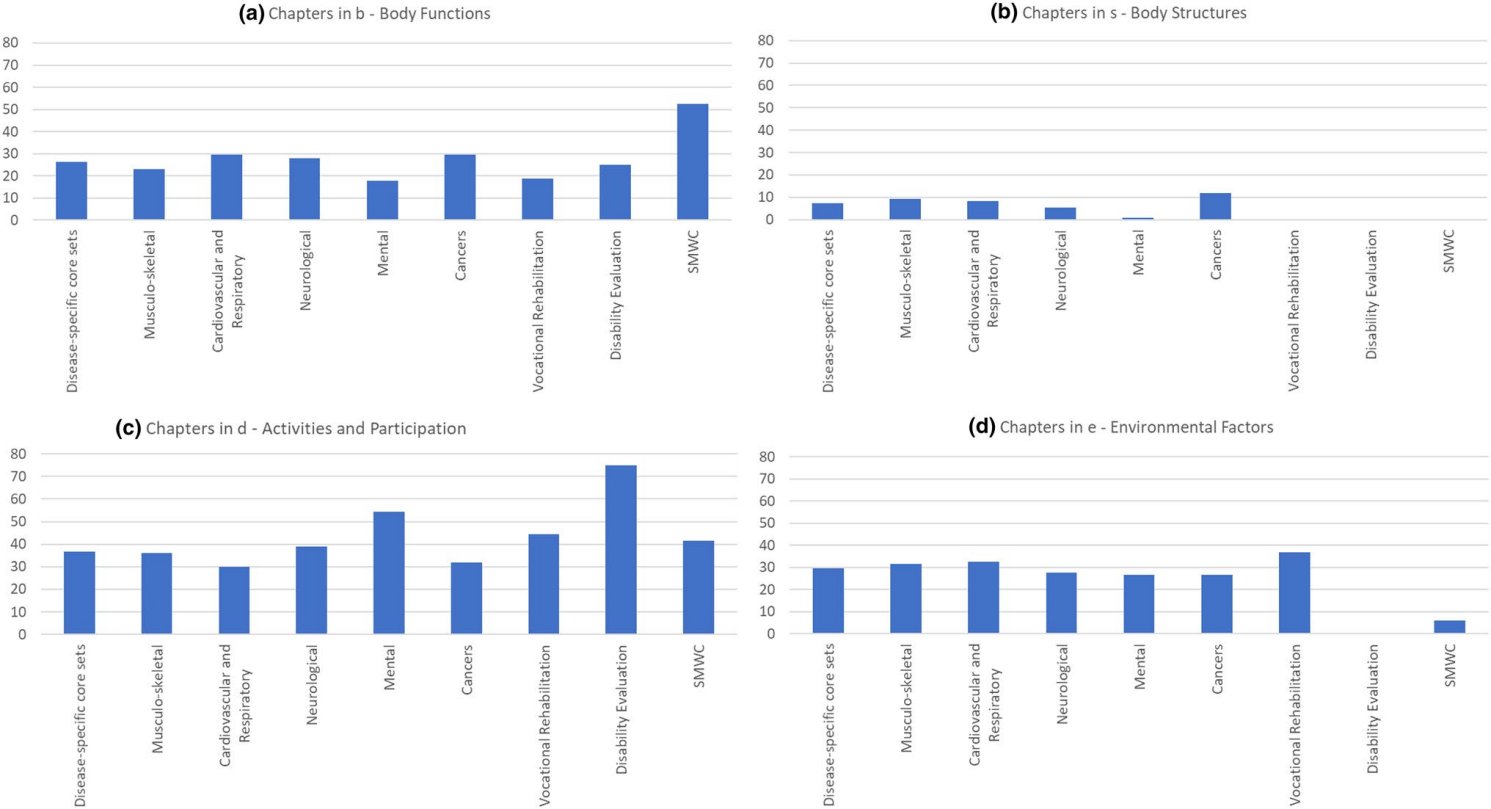
Overview of the distribution of identified ICF categories within the included disease-specific core sets (aggregated by disease group), the work-related core sets and the SMWC over the ICF classifications

The distribution of ICF categories across the ICF components in the SMWC differs from the distribution across disease-specific core sets, with 52.5% from Body Functions, 41.4% from Activities and Participation, and 6.1% from Environmental factors. No categories from the Body Structures are included, see Fig. 3. ICF categories with relative frequencies above 70% are in the Body Functions (N = 6), Activities and Participation (N = 14), and Environmental factors (N = 11), see Text Box 1. When comparing the content of the SMWC with the disease specific core sets on Chapter level, we see overlap in 10 ICF categories with high relative frequencies (> 70%) that are included in most disease specific core sets and the SMWC. Of these, four categories are from the Body functions and six from the Activities and Participation component, see Table 3. Highly frequent ICF categories in the disease specific core sets that are not included in the SMWC are related to social factors, e.g. friends, family and colleagues, factors related to taking care of oneself, e.g. washing, eating, caring for body parts, doing housework, and related to health professionals and systems.

Text Box 2: Disease-Specific Core Sets *Musculoskeletal conditions* (MU), N = 7: Ankylosing spondylitis [32], Chronic widespread pain [33], Low back pain [34], Osteoporosis [35], Osteoarthritis [36], Rheumatoid arthritis [38], Acute arthritis [37]

*Cardiovascular and Respiratory conditions* (CR), N = 6: Stroke [40], Chronic ischemic heart disease [39], Diabetes mellitus [41], Obesity [42], Obstructive pulmonary disease [43], Lymphedema [44]

*Neurological conditions* (N), N = 5: Multiple sclerosis [46], Traumatic brain injury [49], Spinal cord injury [45], Guillain Barré Syndrome [48], Sleep disorder [47]

*Mental conditions* (M), N = 4: Mental disorders [51], Bipolar disorders [50], Depression [52] and Schizophrenia [53], and

*Cancers* (C), N = 3: Head and neck cancer [56], Breast cancer [54], Cancer survivors [55]

Six disease-specific core sets (Attention deficit hyperactivity disorder [57], Autism spectrum disorder [58], Hand Conditions [62], Inflammatory bowel disease [61], Hearing loss [59], and Vertigo, dizziness and balance disorders [60] could not be grouped into these disease groups and were excluded from further analysis

## Discussion

The aim of this study was to examine the content validity of the SMWC by comparing its content with ICF core sets. Comparison of the SMWC with the included work-related and disease specific core sets showed that the SMWC covers most of the relevant items on Body functions and Activities and Participation, however, most of the Environmental factors were lacking.

The relative strong focus on Body functions and Activities and Participation level may be due to the legal context in which the SMWC was developed and used. The SMWC was developed to provide a holistic view of work capacity, including medical history taking and attention to activity limitations and participation restrictions, influencing this capacity [8, 9]. Because of the legal constraints, the assessment is highly protocolized, leaving limited room to take personal and environmental factors into account. This might explain the scarse inclusion of these additional factors in the SMWC. When the outcomes are to be used to provide a holistic assessment of a persons’ residual work capacity and what is needed to find a good jobmatch, the content of the SMWC may therefore not be comprehensive enough. In line with the ICF framework, a dynamic interaction between health and personal and environmental factors are likely to have a direct or indirect influence on a persons’ work capacity [20, 21].

Work-related factors included in the SMWC are in particular assessing the physical work environment (e.g. heat, sound, air quality) and physical job demands, i.e. work endurance, working hours, and level of work exertion. Factors on psychosocial job demands (e.g. job content, decision authority, supervisor and colleagues support) are lacking. With the purpose of jobmatching in mind, additional environmental factors of the work place were included during the SMWC development, additional to the ICF. These factors are based on the content of currenty used methods for work capacity assessments, and relate to, for example work endurance [23]. However, to achieve a jobmatch, not only information about the person and hypothetical workplace factors are needed, but also knowledge about the physical and psychosocial job demands. Since the importance of work-related factors in the assessment of work capacity has long been recognised [22, 23], and that both physical and psychosocial job demands are predictors for work participation [24, 25], it is strongly recommended to extent the SMWC with this type of work characteristics in the work capacity assessment.

Highly frequent social factors, e.g. friends and family, and factors related to taking care of oneself, e.g. washing, eating, caring for body parts, doing housework, illustrate that ICF categories related to the social context might also be relevant to include in the SMWC. This is in line with findings of a recent systematic review showing the relevance of including the social context for work capacity. They concluded that several cognitive behavioural factors of significant others (like friends or family) can facilitate or hinder work participation [26]. When asked, insurance physicians also considered the context of community life, social life and civic life in addition to disease related factors and functions and structures as important factors for work capacity assessments [65].

## Strengths and Limitations

A strength of this study is the use of ICF core sets in examining the content validity of the SMWC, which is in line with recent recommendations by the WHO and others to use the ICF in work disability assessments [22, 27]. Using the ICF framework to evaluate the content validity of the SMWC is a strong and novel approach and allows for a more structured assessment in comparison to expert judgements [14]. The ICF framework provides a holistic view of the person and provides a unified language for expressing these assessments, and core-set development is often standardized and published in peer reviewed publications. An additional strength is the systematical approach in identifying ICF core sets in the literature.

Some limitations should also be reported. The ICF does not operationalize personal factors and lacks specific work-related environmental factors [28–31]. Information about the work context or personal factors might provide valuable information relevant for work capacity evaluation as they possibly act as barriers or facilitators for work participation and are currently not included in our overview. Second, there is criticism regarding the development of core sets as they have a biomedical connotation, while the aim of the ICF is a biopsychosocial approach [15].

## Implications for Research and Practice

The SMWC was developed to guide the social security experts in taking a biopsychosocial approach when creating an overview of a person’s work capacity and what is needed to find a good jobmatch. However, we showed that the instrument still has a focus on Body functions and Activities and Participation, and could be further developed by including additional factors to take into account the home situation (e.g. attitudes and relationships with friends and family), personal care (e.g. washing and doing housework), and workplace factors. Comparisons with disease specific core sets showed additional blind spots in the SMWC content. Further research could also focus on a more tailored use of the SMWC for specific diseases or underlying illness. The content comparisons with the disease specific core sets could therefore be a starting point for selection of relevant content. In addition, more research is needed to identify additional items in particular focussing on the work context, i.e. the implications of functioning problems for work opportunities, the barriers to participation in work, and the workplace adjustments or interventions required to overcome these barriers and achieve a good jobmatch. Work endurance, dealing with different types of working hours, level of exertion, estimating own options, overseeing the consequences of own actions, and achieving workpace are some examples of work related items that are found in the SMWC and not present in the ICF framework [51] and therefore also not identified in the core sets of our review. Additionally, aspects of the psychosocial work environment are important factors for finding a good jobmatch. It is therefore recommended to add these factors to the SMWC and possibly to the ICF framework, see also table S2. To further develop and improve precision and practical use of the SMWC, tailored subsets of the instrument should be identified together with insurance physicians and labor experts, combined with existing literature on barriers and facilitators in the work context or personal factors in various disease groups.

## Conclusion

The SMWC content seems relevant, but needs to be more comprehensive for the purpose of use in work capacity assessments, as it has a relatively strong focus on body functions and activities and participation. To better achieve it’s goal in taking a biopsychosocial approach when creating an overview of a person’s work capacity and what is needed to find a good jobmatch, it is recommended to extend the instrument by adding personal and environmental factors, such as social factors and domestic factors, as well as more specific work related factors. To improve the use of the SMWC in practice, it is recommended to select the relevant disease-specific categories out of the comprehensive instrument, to aid a tailored use of the instrument.

## Electronic supplementary material

Below is the link to the electronic supplementary material.

Supplementary file1 (DOCX 46 kb)
