# Supplementary material for: Content Validation of a Practice-Based Work Capacity Assessment Instrument Using ICF Core Sets
Source: J Occup Rehabil. 2020 Aug 16;31(2):293–315. doi: 10.1007/s10926-020-09918-7 (PMC8172508; doi:10.1007/s10926-020-09918-7)
Supplement: Supplementary file 1 — Supplementary file1 (DOCX 46 kb) [file 10926_2020_9918_MOESM1_ESM.docx]

**Supplemental material belonging to** Sengers et al. (2020) Content Validation of a Practice-Based Work Capacity Assessment Instrument Using ICF Core Sets, Journal of Occupational Rehabilitation
**DOI :** 10.1007/s10926-020-09918-7

## Supplementary Table S1: Search strategy

| Web of science | Medline | PsycInfo |
| --- | --- | --- |
| ((‘disability evaluation’ OR ‘work ability’ OR ‘work functioning’ OR ‘work capacity’)  AND Topic=(assessment OR evaluation OR develop* OR instrument* OR questionnaire*) OR (‘core set’ OR ‘core sets’))  AND  (icf OR ‘international classification’) | ((‘core set’ OR ‘core sets’) AND (icf OR international classification)) OR (((MH ‘Disability Evaluation+’) OR ‘disability evaluation’) OR ((MH ‘Work Capacity Evaluation’) OR ‘work capacity’) OR ((work ability) OR (work functioning)) AND  (icf OR international classification)  AND  (assessment OR evaluation OR develop* OR instrument* OR questionnaire*)) | ((DE ‘Disability Evaluation’ OR (‘disability evaluation’ OR ‘work capacity’ OR ‘work functioning’ OR ‘work ability’)) AND  (assessment OR evaluation OR develop* OR instrument* OR questionnaire*) OR (‘core set’ OR ‘core sets’))  AND  (icf OR ‘international classification’) |

## Supplementary Table S2: Full description of SMWC

| **Body Functions** | |  |  |  |  |  |  |
| --- | --- | --- | --- | --- | --- | --- | --- |
| **b1** | **Mental functions** | b167 | Mental functions of language | **b4** | **Functions of the cardiovascular, hematological, immunological and respiratory systems** | **b6** | **Genitourinary and reproductive functions** |
| b110 | Consciousness functions | b172 | Calculation functions | b410 | Heart functions | b620 | Urination functions |
| b114 | Orientation functions | b176 | Mental functions of language | b415 | Blood vessel functions | **b7** | **Neuromusculoskeletal and movement-related functions** |
| b117 | Intellectual functions | **b2** | **Sensory functions and pain** | b420 | Blood pressure functions | b710 | Mobility of joint functions |
| b122 | Global psychosocial functions | b210 | Seeing functions | b430 | Hematological system functions | b715 | Stability of functions |
| b125 | Dispositions and intra-personal functions | b230 | Hearing functions | b435 | Immunological system functions | b730 | Muscle power functions |
| b126 | Temperament and personality functions | b235 | Vestibular functions | b440 | Respiration functions | b735 | Muscle tone functions |
| b130 | Energy and drive functions | b240 | Sensations associated with hearing and vestibular function | b445 | Respiratory muscle function | b740 | Muscle endurance functions |
| b140 | Attention functions | b260 | Proprioceptive functions | b450 | Additional respiratory functions | b750 | Motor reflex function |
| b144 | Memory functions | b265 | Touch function | b455 | Excersice tolerance functions | b755 | Involuntary movement reaction functions |
| b147 | Psychomotor functions | b270 | Sensory functions related to temperature and other stimuli | **b5** | **Functions of the digestive, metabolic, endocrine systems** | b760 | Control of voluntary movement functions |
| b152 | Emotional functions | b280 | Sensation of pain | b525 | Defecation functions | b765 | Involuntary movement functions |
| b156 | Perceptual functions | **b3** | **Voice and speech functions** | b540 | General metabolic functions | **b8** | **Functions of the skin and related functions** |
| b160 | Thought functions | b310 | Voice functions | b550 | Thermoregulatory functions | b820 | Repair functions of the skin |
| b163 | Basic cognitive functions | b320 | Articulation functions | b555 | Endocrine gland functions | b810 | Protective functions of the skin |
| b164 | Higher level cognitive functions | b330 | Fluency and rhythm of speech functions |  |  |  |  |
| **Activities and participation** | |  |  |  |  |  |  |
| **d1** | **Learning and applying knowledge** | d2202 | Undertaking multiple tasks independently | **d3** | **Communication** | d4105 | Bending |
| d110 | Watching | d2203 | Undertaking multiple tasks in a group | d310 | Communicating with-receiving- spoken messages | *d4108a* | *Changing basic body position, twist* |
| d115 | Listening | d2204 | Completing multiple tasks independently | d315 | Communicating with-receiving- non-verbal messages | *d4108b* | *Changing basic body position, head movement* |
| d1201 | Other purposeful sensing | d2205 | Completing multiple tasks in a group | d325 | Communicating with-receiving-written messages | d415 | Maintaining a body position |
| d155 | Acquiring skills | *d2208a* | *Undertaking multiple tasks, to structure* | d330 | Speaking | d4151 | Maintaining a squatting position |
| d159 | Basic learning other specified, to remind | *d2208b* | *Undertaking multiple tasks, to set priorities* | d335 | Producing non-verbal messages | d4152 | Maintaining a kneeling position |
| d160 | Focusing attention | d230 | Carrying out daily routine | d340 | Producing messages in formal sign language | d4153 | Maintaining a sitting position |
| d166 | Reading | d240 | Handling stress and other psychological demands | d345 | Writing messages | d4154 | Maintaining a standing position |
| d170 | Writing | d2500 | Managing one's own behavior, accepting novelty | *d349a* | *Communication-producing, expressing own feelings* | d4155 | Maintaining head position |
| d172 | Calculating | d2508 | Managing one’s own behavior, asking for help | **d4** | **Mobility** | *d4158a* | *Maintaining a body position, bent posture* |
| **d2** | **General tasks and demands** | *d298a* | *General tasks and demands, estimating own options* | d4101 | Changing basic body position, squatting | *d4158b* | *Maintaining a body position, twisted posture* |
| d2102 | Undertaking a single task independently | *d298b* | *General tasks and demands, overseeing the consequences of own actions* | d4102 | Changing basic body position, kneeling | *d4158c* | *Maintaining a body position, arm in certain posture* |
| d2103 | Undertaking a single task in a group | *d298c* | *General tasks and demands, achieving work pace* | d4103 | Changing basic body position, sitting | d430 | Lifting and carrying objects |
|  |  |  |  |  |  |  |  |
| **Activities and participation (continued)** | | |  |  |  |  |  |
| d4301 | Carrying in the hands | d540 | Dressing |  |  |  |  |
| d4302 | Carrying in the arms | d570 | Looking after one’s health |  |  |  |  |
| d4303 | Carrying on shoulders, hip and back | d571 | Self-care, safety |  |  |  |  |
| d440 | Fine hand use | **d7** | **Interpersonal interactions and relationships** |  |  |  |  |
| d4408 | Fine hand use, control of keyboard and or mouse | d7100 | Respect and warmth in relationships |  |  |  |  |
| d445 | Hand and arm use | d7102 | Tolerance in relationships |  |  |  |  |
| d4450 | Pulling | d7103 | Criticism in relationships |  |  |  |  |
| d4451 | Pushing | d7105 | Physical contact in relationships |  |  |  |  |
| d4452 | Reaching | d720 | Complex interpersonal interactions |  |  |  |  |
| d4453 | Flip or rotate the hands or arms | d7208 | Complex interpersonal interactions, handling conflicts |  |  |  |  |
| d450 | Walking | d730 | Relating with strangers |  |  |  |  |
| d4551 | Climbing | d7400 | Relating with persons in authority |  |  |  |  |
| d446 | Walking and moving, fine foot use | **d8** | **Major life areas** |  |  |  |  |
| d470 | Using transportation | *d859a* | *Work and employment,*  *work endurance* |  |  |  |  |
| d475 | Driving | *d859b* | *Work and employment, dealing with different types of working hours* |  |  |  |  |
| **d5** | **Self-care** | *d859c* | *Work and employment, level of exertion* |  |  |  |  |
| d510 | Washing oneself |  |  |  |  |  |  |
| d520 | Caring for body parts |  |  |  |  |  |  |
| d530 | Toileting |  |  |  |  |  |  |
| **Environmental Factors** | |  |  |  |  |  |  |
| **e1** | **Products and technology** | **e2** | **Natural environment and human-made changes to environment** |  |  |  |  |
| *e1358a* | *Products and technology for employment, exposure to specific substances* | *e2250a* | *Temperature, heat* |  |  |  |  |
| *e1358b* | *Products and technology for employment, wear of protective equipment* | *e2250b* | *Temperature, cold* |  |  |  |  |
|  |  | e2254 | Wind |  |  |  |  |
|  |  | e240 | Light |  |  |  |  |
|  |  | e250 | Sound |  |  |  |  |
|  |  | e255 | Vibration |  |  |  |  |
|  |  | e260 | Air quality |  |  |  |  |
|  |  |  |  |  |  |  |  |

Codes ending with 8 (other specified) and 9 (unspecified) and codes ending with a, b or c are additions or refomulations on the ICF Framework used in SMWC and presented in *italics*.

## Supplementary Table S3: Overview of the distribution of identified ICF categories within the included disease-specific core sets (aggregated by disease group), the work-related core sets and the SMWC over the ICF classifications and chapters

|  |  |  | Disease-specific core sets |  | Musculo-skeletal  conditions | Cardiovascular and Respiratory  conditions | Neurological  conditions | Mental  conditions | Cancers | Vocational Rehabilitation | Disability  Evaluation | SMWC |
| --- | --- | --- | --- | --- | --- | --- | --- | --- | --- | --- | --- | --- |
|  |  |  | N= 25 |  | N=7 | N=6 | N=5 | N=4 | N=3 | N=1 | N=1 | N=1 |
| Chapters in b - Body Functions | |  | 26.4% |  | 23.0% | 29.4% | 28.0% | 17.9% | 29.5% | 18.9% | 25.0% | 52.5% |
| b1 | Mental functions |  | 28.7% |  | 27.8% | 24.1% | 26.5% | 69.6% | 26.9% | 52.9% | 20.0% | 30.8% |
| b2 | Sensory functions and pain |  | 11.8% |  | 10.4% | 10.3% | 13.9% | 4.3% | 15.4% | 23.5% | 20.0% | 15.4% |
| b3 | Voice and speech functions |  | 3.3% |  | 0.0% | 2.8% | 6.0% | 4.3% | 3.8% | 0.0% | 0.0% | 5.8% |
| b4 | Functions of the cardiovascular, haematological, immunological and respiratory systems |  | 14.5% |  | 12.2% | 22.8% | 12.7% | 2.2% | 9.6% | 5.9% | 20.0% | 17.3% |
| b5 | Functions of the digestive, metabolic and endocrine systems |  | 8.1% |  | 1.7% | 12.4% | 7.8% | 8.7% | 9.6% | 0.0% | 0.0% | 7.7% |
| b6 | Genitourinary and reproductive systems |  | 8.1% |  | 5.2% | 9.7% | 7.8% | 6.5% | 11.5% | 0.0% | 0.0% | 1.9% |
| b7 | Neuromusculoskeletal and movement-related functions |  | 22.0% |  | 42.6% | 12.4% | 22.3% | 4.3% | 13.5% | 11.8% | 40.0% | 17.3% |
| b8 | Functions of the skin and related structures |  | 3.5% |  | 0.0% | 5.5% | 3.0% | 0.0% | 9.6% | 5.9% | 0.0% | 3.8% |
|  |  |  | 100% |  | 100% | 100% | 100% | 100% | 100% | 100% | 100% | 100% |

|  |  |  |  |  |  |  |  |  |  |  |  |  |
| --- | --- | --- | --- | --- | --- | --- | --- | --- | --- | --- | --- | --- |
| Chapters in s - Body Structures | |  | 7.4% |  | 9.4% | 8.3% | 5.4% | 0.8% | 11.9% | 0.0% | 0.0% | 0.0% |
| s1 | Structures of the nervous system |  | 9.8% |  | 2.1% | 14.6% | 18.8% | 0.0% | 4.8% | 0.0% | 0.0% | 0.0% |
| s2 | Eye, ear and related structures |  | 3.5% |  | 8.5% | 2.4% | 0.0% | 0.0% | 0.0% | 0.0% | 0.0% | 0.0% |
| s3 | Structures involved in voice and speech |  | 4.2% |  | 0.0% | 0.0% | 9.4% | 0.0% | 14.3% | 0.0% | 0.0% | 0.0% |
| s4 | Structures cardiovascular, immunological or respiratory system |  | 14.0% |  | 8.5% | 22.0% | 9.4% | 0.0% | 19.0% | 0.0% | 0.0% | 0.0% |
| s5 | Structures of the digestive, metabolic and endocrine systems |  | 7.0% |  | 0.0% | 17.1% | 3.1% | 0.0% | 9.5% | 0.0% | 0.0% | 0.0% |
| s6 | Structures related to the genitourinary and reproductive systems |  | 4.2% |  | 0.0% | 7.3% | 6.3% | 0.0% | 4.8% | 0.0% | 0.0% | 0.0% |
| s7 | Structures related to movement |  | 49.7% |  | 74.5% | 29.3% | 46.9% | 50.0% | 38.1% | 0.0% | 0.0% | 0.0% |
| s8 | Skin and related structures |  | 7.7% |  | 6.4% | 7.3% | 6.3% | 50.0% | 9.5% | 0.0% | 0.0% | 0.0% |
|  |  |  | 100% |  | 100% | 100% | 100% | 100% | 100% | 0% | 0% | 0% |
|  |  |  |  |  |  |  |  |  |  |  |  |  |
| Chapters in d - Activities and Participation | |  | 36.8% |  | 35.9% | 29.8% | 39.0% | 54.5% | 31.8% | 44.4% | 75.0% | 41.4% |
| d1 | Learning and applying knowledge |  | 7.6% |  | 1.7% | 5.4% | 10.0% | 13.6% | 5.4% | 20.0% | 26.7% | 19.5% |
| d2 | General tasks and demands |  | 7.5% |  | 5.0% | 7.5% | 7.4% | 10.7% | 8.9% | 10.0% | 13.3% | 14.6% |
| d3 | Communication |  | 5.2% |  | 0.6% | 6.1% | 7.4% | 8.6% | 5.4% | 10.0% | 6.7% | 17.1% |
| d4 | Mobility |  | 24.1% |  | 36.7% | 28.6% | 21.6% | 5.0% | 14.3% | 25.0% | 46.7% | 24.4% |
| d5 | Self-care |  | 14.8% |  | 16.7% | 14.3% | 12.6% | 12.1% | 21.4% | 7.5% | 0.0% | 12.2% |
| d6 | Domestic life |  | 11.1% |  | 15.0% | 10.9% | 10.0% | 9.3% | 10.7% | 0.0% | 0.0% | 0.0% |
| d7 | Interpersonal interactions and relationships |  | 11.4% |  | 7.8% | 10.2% | 10.4% | 17.1% | 19.6% | 7.5% | 6.7% | 9.8% |
| d8 | Major life areas |  | 12.0% |  | 10.0% | 10.9% | 15.2% | 15.7% | 7.1% | 20.0% | 0.0% | 2.4% |
| d9 | Community, social and civic life |  | 6.3% |  | 6.7% | 6.1% | 5.6% | 7.9% | 7.1% | 0.0% | 0.0% | 0.0% |
|  |  |  | 100% |  | 100% | 100% | 100% | 100% | 100% | 100% | 100% | 100% |
|  |  |  |  |  |  |  |  |  |  |  |  |  |
| Chapters in e - Environmental Factors | |  | 29.5% |  | 31.7% | 32.5% | 27.7% | 26.8% | 26.7% | 36.7% | 0.0% | 6.1% |
| e1 | Products and technologies |  | 20.1% |  | 23.9% | 20.0% | 20.7% | 10.1% | 14.9% | 24.2% | 0.0% | 16.7% |
| e2 | Natural environment and human-made changes to environment |  | 5.5% |  | 5.0% | 5.0% | 6.1% | 5.8% | 2.1% | 12.1% | 0.0% | 83.3% |
| e3 | Support and relationships |  | 24.1% |  | 23.9% | 25.0% | 22.0% | 24.6% | 27.7% | 21.2% | 0.0% | 0.0% |
| e4 | Attitudes |  | 26.1% |  | 28.3% | 25.0% | 23.8% | 34.8% | 21.3% | 12.1% | 0.0% | 0.0% |
| e5 | Services, systems and policies |  | 24.3% |  | 18.9% | 25.0% | 27.4% | 24.6% | 34.0% | 30.3% | 0.0% | 0.0% |
|  |  |  | 100% |  | 100% | 100% | 100% | 100% | 100% | 100% | 0% | 100% |
| **SMWC**=**S**ocial **M**edical **W**ork **C**apacity instrument. The group 'Other health conditions' (N=6) of the disease-specific core sets is not included in this table due to diversity within the group.  The total number of ICF categories are summed for each classification or chapter and divided by the total number of ICF categories in the particular classification or chapter and presented in percentages. | | | | | | | | | | | | |

## Supplementary Figure S1: Overlap between SMWC and the two work-related core sets on Body Functions, Activities and Participation, and Environmental Factors

SMWC = Social Medical Work Capacity instrument
